# Species-Selective Killing of Bacteria by Antimicrobial Peptide-PNAs

**DOI:** 10.1371/journal.pone.0089082

**Published:** 2014-02-18

**Authors:** Madhav Mondhe, Ashley Chessher, Shan Goh, Liam Good, James E. M. Stach

**Affiliations:** 1 School of Biology, Newcastle University, Newcastle upon Tyne, United Kingdom; 2 Department of Pathology and Infectious Diseases, Royal Veterinary College, University of London, London, United Kingdom; Florida International University, United States of America

## Abstract

Broad-spectrum antimicrobials kill indiscriminately, a property that can lead to negative clinical consequences and an increase in the incidence of resistance. Species-specific antimicrobials that could selectively kill pathogenic bacteria without targeting other species in the microbiome could limit these problems. The pathogen genome presents an excellent target for the development of such antimicrobials. In this study we report the design and evaluation of species-selective peptide nucleic acid (PNA) antibacterials. Selective growth inhibition of *B. subtilis*, *E. coli*, *K. pnuemoniae* and *S. enterica* serovar Typhimurium in axenic or mixed culture could be achieved with PNAs that exploit species differences in the translation initiation region of essential genes. An *S*. Typhimurium-specific PNA targeting *fts*Z resulted in elongated cells that were not observed in *E. coli*, providing phenotypic evidence of the selectivity of PNA-based antimicrobials. Analysis of the genomes of *E. coli* and *S*. Typhimurium gave a conservative estimate of >150 PNA targets that could potentially discriminate between these two closely related species. This work provides a basis for the development of a new class of antimicrobial with a tuneable spectrum of activity.

## Introduction

Treatment of bacterial infections with antimicrobial drugs has been clinically effective for over six decades. However, the rise of antimicrobial resistance threatens to limit options for the treatment of life-threatening microbial diseases [1]. Broad-spectrum antibiotics allow empirical usage for the rapid treatment of fulminate microbial infections, but have been shown to disrupt the microbiome, which can result in colonization of pathogenic microbes [2], [3], increase susceptibility to disease [4], [5], and select for resistance mechanisms in non-target species that are readily transferred to pathogenic species [6], [7]. Recent findings demonstrating the importance of the microbiome in host health have led to a growing interest in possibilities to use pathogen or microbiota-targeted therapies to eliminate individual strains of single species [8]. One approach is silencing of essential genes using antisense mechanisms. Exogenously delivered antisense DNA oligonucleotides, designed to bind to specific mRNA sequences, have been demonstrated to be effective against bacterial targets [9], [10]. Peptide Nucleic Acid (PNA) and Phosphorodiamidate Morpholino (PMO) oligomers targeted to the translation initiation region (TIR) of essential mRNAs are bactericidal and have been successfully applied to a number of different species [11]–[14]. Bactericidal PNAs/PMOs are typically 10 bp in length and are more sensitive to target mismatches than equivalent DNA oligonucleotides [15], properties that make them highly suited to species discrimination.

In this study we tested the hypothesis that the selective binding properties of peptide-PNA antimicrobials can be exploited to selectively target certain species in mixed culture based on sequence differences in the translation initiation region of essential genes. A representative Gram-positive bacterium (*Bacillus subtilis*) and three Gram-negative bacteria (*Klebsiella pneumoniae*, *Salmonella* Typhimurium and *Escherichia coli*) were chosen as a mix-species model in this study. We report, for the first time, that peptide-PNAs can form the basis of a single class of antimicrobial with a tuneable spectrum of activity.

## Results

### Peptide-PNA mediated growth inhibition and species-selectivity

The four bacterial species were chosen as they have all been reported to be susceptible to antisense antibiotics [12], [14]–[16]. The parameters used in the design of species-selective peptide-PNAs resulted in a number of potential gene targets that could be used for species-selective growth inhibition. The following criteria were used for the design of species-selective peptide PNAs: 1) target gene is essential and homologues are present in all four species used in this study; 2) the translation initiation region (TIR) of the mRNA had at least two base-pair differences between species (see below); 3) the TIR sequence was amenable to the design of peptide-PNAs with low melting temperatures; 4) where possible, off-target sites within and between species were not in the TIRs of essential genes; and 5) evidence that gene silencing of the target and/or inhibition of its cognate protein is growth inhibitory. We have previously shown that *mur*A and *fts*Z are sensitive targets for peptide-PNA mediated growth inhibition [17], both genes were identified in this study as potential targets for species-selective peptide-PNAs, and thus, were selected for further study. Two base-pair mismatches were selected, as PNAs with one base-pair mismatch may bind to the target, but with reduced affinity [15]; peptide-PNAs with one base-pair mismatch to their target sites have shown approximately 33% increase in MIC (Liam Good, unpublished). This is in agreement with the design parameters suggest by Dryselius et al. [18] in which they suggest 1-bp mismatches within the TIR of off-target genes should be avoided.

**Figure 1:**
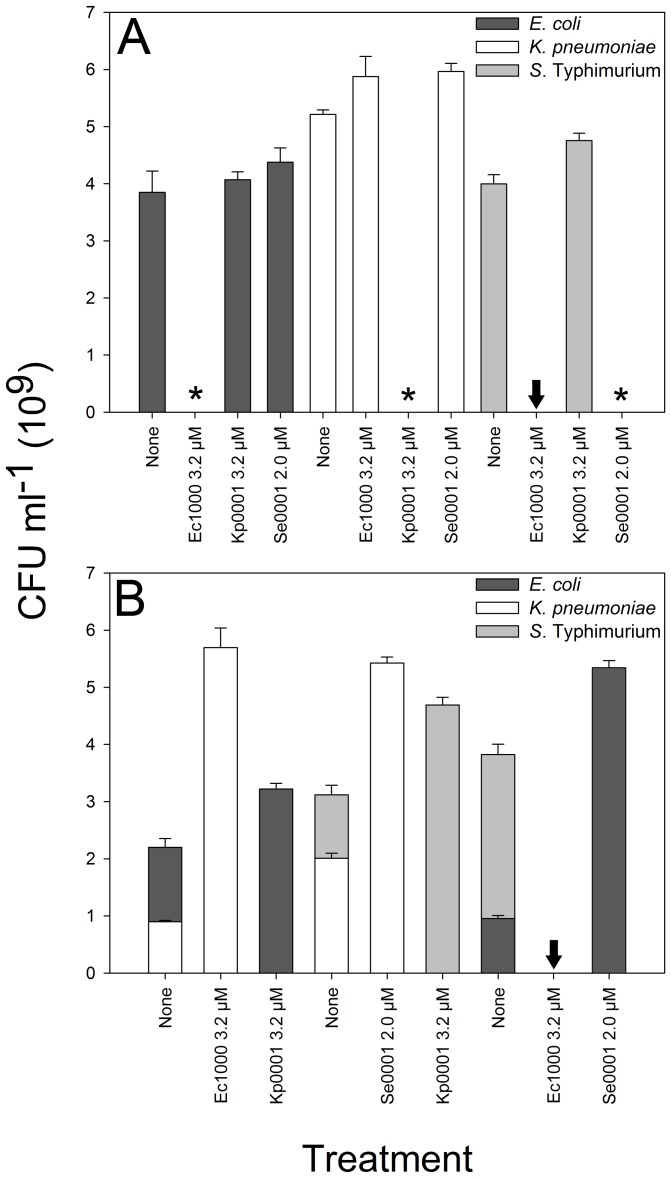
Species-selective antibacterial peptide-PNAs in axenic and two-species mixed culture. *E. coli* (dark grey), *K. pneumoniae* (white) and *S*. Typhimurium (light grey). All cultures were incubated for 16 hrs. A) axenic cultures of the species were treated with *E. coli*- specific Ec1000 at 3.2 µM, *K. pneumoniae*-specific Kp0001 at 3.2 µM and *S*. Typhimurium-specific Se0001 at 2.0 µM. Asterisks indicate species-selective growth inhibition of *E. coli*, *K. pneumoniae* and *S*. Typhimurium respectively. B) Two-species mixed cultures treated with peptide-PNAs as above. The control cultures show the relative proportion of the two species without treatment, the two treatments to the left of the control represent the same mixed culture treated with a peptide-PNA. Black arrows indicate non species-selective growth inhibition of *S*. Typhimurium by Ec1000. Error bars are standard error for biological replicates (*n* = 3).

The peptide-PNAs designed in this study were assayed for their antibacterial activity against both target and non-target species. The previously reported En108 peptide-PNA (called Ec108 in [17]) was used as a control to test the feasibility of species-selectivity at a broad taxonomic level; *E. coli, K*. *pneumoniae* and *S*. Typhimurium (Gram-negative, Enterobacteriaceae) have identical *acp*P TIRs and thus all three species should be susceptible to En108, while the *acp*P TIR of *Bacillus subtilis* (Gram-positive, Bacillaceae) has six base-pair mismatches and should be resistant to En108. Antibacterial assays with En108 proved this to be the case; En108 had an MIC of 1.2, 0.4 and 0.3 µM for *E. coli, K*. *pneumoniae* and *S.* Typhimurium respectively (Table 1), and had no detectable antibacterial activity against *B. subtilis* at concentrations of up to 20 µM (data not shown). Similarly, the species-selective PNAs for *B. subtilis*, *K. pneumoniae* and *S*. Typhimurium were only antibacterial to the intended species (Figure 1A). The *E. coli*-selective Ec1000 was unexpectedly cross-reactive with *S*. Typhimurium (discussed below). In all cases treatment with non-specific PNAs resulted in increased, but statistically insignificant (standard error P>0.05), growth. Table 2 shows the analysis of potential binding sites within the genomes of the target species. Of note is the difference in MIC between *E. coli*, *K. pneumoniae* and *S*. Typhimurium when treated with the *acp*P-targeting En108; the MIC of this PNA was 3 and 4 fold less in *K. pneumoniae* and *S*. Typhimurium, respectively (Table 1). Analysis of the binding sites of En108, Kp0001 and Se0001 in the genomes of these species revealed that En108 likely binds in the TIR region of other essential genes in *K. pneumoniae* (*muk*F and *rib*H) and *S*. Typhimurium (*yhh*M) each with a 1 bp mismatch. This could account for the decreased MIC in these species, however the relationship is not straightforward as Se0001 is predicted to bind in the TIR of at least three other genes determined to be essential in *E. coli* and *S*. Typhimurium (*hem*K, *lnt* and *rlu*A) and has an MIC equivalent to that of En108 in *E. coli* (Table 1). Furthermore, there is no obvious relationship between the MIC of a peptide-PNA and the number of off-targets in the genome of the target species, including those that bind in the TIRs of both essential and non-essential genes (Table 2). Reasons for the possible differences between the MICs of the different peptide-PNAs are discussed below.

**Table 1:** Properties of peptide-PNAs used in this study.

Name^a^	Target	Sequence	Minimum Inhibitory Concentration (µM)				Expected specificity^b^	Target site	*T*_m_ d	Reference
			*B. subtilis*	*E. coli*	*K. pneumonia*	*S.* Typhimurium				
Bs0001	*fts*Z	(KFF) _3_K-eg-caacatgcta	4.0	>10	>10	>10	yes	−4 to +6	53.5	This study
En108	*acp*P	(KFF) _3_K-eg-ctcatactct	>10	1.2	0.4	0.3	yes	−5 to +5	41.5	Goh et al. 2009
Ec1000	*mur*A	(KFF) _3_K-eg-ccatttagtt	>10	2.4	>10	3.2	no^e^	−6 to +4	44.0	This study
Kp0001	*mur*A	(KFF) _3_K-eg-tccattgatt	>10	>10	2.5	>10	yes	−5 to +5	46.8	This study
Se0001	*mur*A	(KFF) _3_K-eg-tccattattg	>10	>10	>10	1.2	yes	−5 to +5	43.5	This study
Se0002	*fts*Z	(KFF) _3_K-eg-aacataatct	>10	>10	>10	2.5	yes	−5 to +5	46.1	This study

a Code refers to predicted species-specificity Bs  = *B. subtilis*, Ec  =  *E. coli*, En  =  Enterobacteriaceae, Kp  =  *K. pneumonia*, Se  =  *S. enterica* Typhimurium.

b Indicates if specificity based on bioinformatic prediction was observed.

c Target is shown as positions of nucleotides relative to the start codon.

d Thermal stability of PNA/DNA duplex.

e Ec1000 lacked predicted specificity, see text for details.

**Table 2:** PNA binding site analysis in target species.

Species	PNA	No. off targets^a^	Off Targets within TIR^b^ of gene	
			No. essential (gene)^c^	No. non-essential (gene)
*E. coli*	En108	128	0	11 (*ara*H, *arp*B, *cad*B, *gpp*, *ssu*E, *ugp*Q, *upp*, *yag*W, *yhj*G, *yod*C, *yqj*F)
*K. pneumoniae*	En108	143	2 (*muk*F^d^^,^ e, *rib*H^d^^,^ f)	5 (*gpp*A, kpn_00790, kpn_01124, kpn_04794, *nlp*A)
*S.* Typhimurium	En108	138	1 (*yhh*M^d^)	14 (*dps*, *hyc*E, *pmg*I, STM0566, STM0762, STM1698, STM2137, STM2481, STM3085, STM4218, *thi*J, *upp, ydc*Y, *yga*C)
*E. coli*	Ec1000	201	2 (*lnt* d ^,^ f, *rps*N^d^^,^ f)	11 (*ela*D, *hyp*F, *mrc*A, *pps*R, *rhs*D, *ybf*D, *ydc*C, *yfb*M, *yha*J, *yhh*I, *yjg*H)
*S.* Typhimurium	Se0001	320	6 (*asp*C^f^, *hem*K^d^^,^ e ^,^ f, *lnt* d ^,^ f, *nuo*I^f^, *pur*D^f^, *rlu*A^d^^,^ f)	8 (*fuc*A, *ppi*C, *sod*A, STM2343, STM2903, *yaj*B, *ybd*F, *yeb*B)
*S.* Typhimurium	Se0002	311	0	11 (*cel*C, t0363, t0453, t1718, t2652, t4467, *tdc*E, *umu*C, *yac*K, *yad*I, *yba*B)
*K. pneumoniae*	Kp0001	157	3 (*isp*A^d^^,^ f, *rpsN* d ^,^ f, *top*B^e^)	3 (KPN_02027, KPN_04368, *rha*T)
*B. subtilis*	Bs0001	104	0	4 *(yab*Q, *yee*G, *yru*I, *yvy*E)

a includes sites with ≤ 1base-pair mismatch with PNA.

b Translation Initiation Region.

c identified by BLAST searching of the Database of Essential Genes.

d essential in *E. coli.*

e essential in *S.* Typhimurium.

f essential in other prokaryotes.

ND Not determined.

### Use of species-selective PNAs in mixed culture

**Figure 2:**
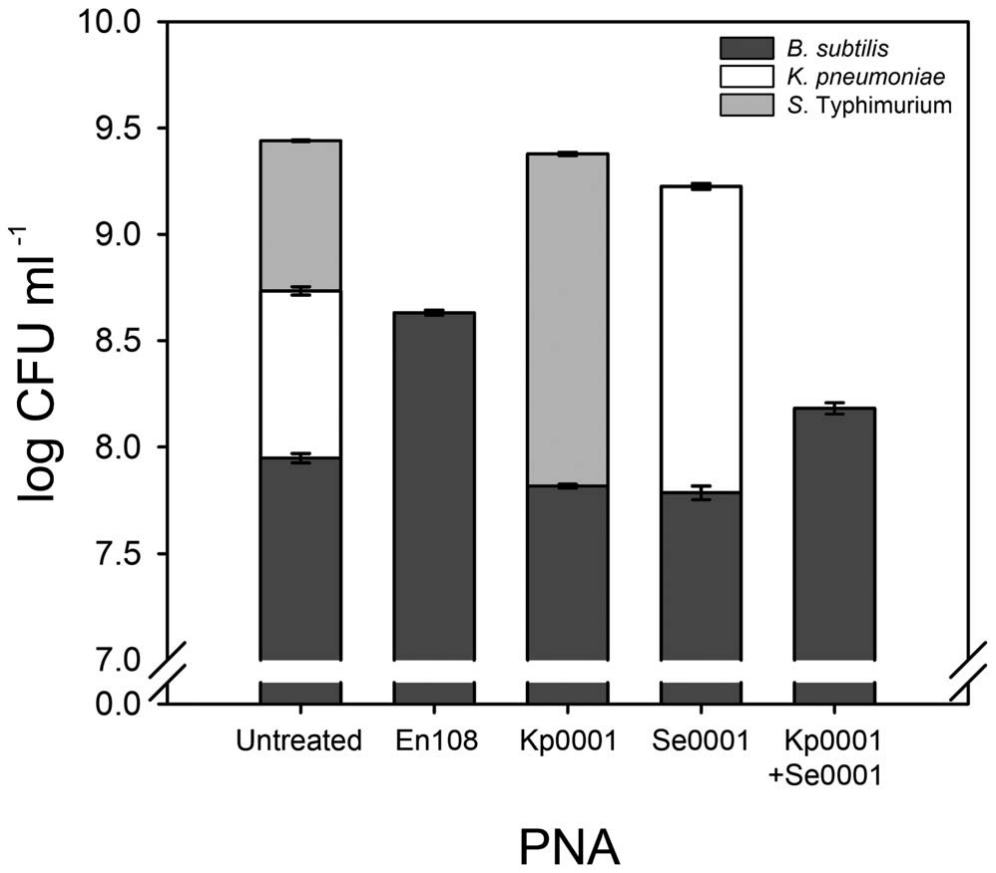
Species-selective antibacterial peptide-PNAs in three-species mixed culture. *B. subtilis* (dark grey), *K. pneumoniae* (white) and *S*. Typhimurium (light grey) in mixed culture were separately treated with Ec108 at 3.5 µM, Kp0001 or Se0001 at 4.5 µM or by combined treatment of Kp0001 and Se0001 both at 4.5 µM. All cultures were incubated for 16 hrs. Selective inhibition of either *K. pneumoniae* or *S*. Typhimurium individually or together, achieved with the peptide-PNAs, could not theoretically be achieved with any combination of the twenty known antimicrobial compounds tested in this study. Error bars as above.

To test the selectivity of peptide-PNAs in mixed culture, we first used reciprocal treatment in two-species culture (Figure 1B). When mixed cultures of *E. coli* and *K. pneumoniae* were treated with 3.2 µM of *E. coli*-selective Ec1000, after 16 hrs of incubation only *K. pneumoniae* was detectable. Untreated control cultures maintained both species throughout the incubation period. Reciprocal treatment of the same mixed culture with 3.2 µM of *K. pneumoniae*-specific Kp0001 showed equivalent selectivity. Species-selective growth inhibition was also observed in mixed culture of *K. pneumoniae* and *S*. Typhimurium treated with 3.2 µM Kp0001 or 2.0 µM Se0001 (Figure 1B). *S*. Typhimurium was successfully removed from mixed culture with *E. coli* when treated with 2.0 µM Se0001, but reciprocal removal of *E. coli* could not be achieved with Ec1000 (see above). Three-species mixed culture of *B. subtilis*, *K. pneumoniae* and *S*. Typhimurium was used to test the possibility of specifically targeting either one or two species, with a single peptide-PNA or a combination of two. Kp0001 and Se0001 both at 4.5 µM were successfully applied to the three-species mixed culture; only *B. subtilis* and *S*. Typhimurium could be detected after 16 hrs incubation with Kp0001, and only *B. subtilis* and *K. pneumoniae* after Se0001 treatment (Figure 2). Treatment of the three-species culture with 3.5 µM En108 resulted in the expected selective growth inhibition of both enteric species, with only *B. subtilis* detectable after 16 hrs. Combined use of Kp0001 and Se0001, at 4.5 µM each was also able to specifically remove *K. pneumoniae* and *S*. Typhimurium from the mixed culture, however there was a significant difference in the final *B. subtilis* CFU count between the En108 and dual Kp0001/Se0001 treated cultures. Mixed culture experiments with four species were not attempted in this study, however the potential of peptide-PNAs as species-selective antibacterial compounds is highlighted by a comparison of all possible species combinations for the four species tested in this study, alongside the antibiotic spectrum of the peptide-PNAs and twenty known antibiotics (Figure S1 in Information S1).

Only peptide-PNAs are capable of species-selective growth inhibition for the three Gram-negative species. For these species, there are six possible outcomes for species-selective antibacterial treatment of two-species mixed cultures. Treatment with the twenty antibiotics assessed in this study (Table S1 in Information S1), could theoretically achieve four of these outcomes (all combinations except those requiring inhibition of *E. coli* or *K. pneumoniae* in combination with *S*. Typhimurium). Use of peptide-PNAs Ec1000, Se0001 and Kp0001 enabled five of the possible outcomes; unexpected cross-reactivity of Ec1000 prevented selective inhibition of *E. coli* in combination with *S*. Typhimurium. However, it is very likely that evaluation of other *E. coli*-selective peptide PNAs would be able to rectify this result (see below). Furthermore, of the known antibiotics, only streptomycin could select between *E. coli* and *S*. Typhimurium. This would not be the case for most strains of *E. coli* as strain DH10B has an *rpsL* mutation that confers resistance to streptomycin. Unlike the peptide-PNAs, in mixed culture experiments with three species (Figure 2), no combination of the known antibiotics would be able to selectively kill any of the Gram-negative species tested without killing *B. subtilis*.

### Peptide-PNA mediated discrimination of *E. coli* and *S*. Typhimurium

The observation that Ec1000 was antibacterial to the non-target *S*. Typhimurium led us to use a comprehensive genomic analysis to determine the number of potential targets that could be used to design peptide-PNAs that would discriminate between these two closely related species. For the purposes of defining potential discriminatory target sites, we allowed for 1 bp mismatch difference between the target site of *S*. Typhimurium and *E. coli*; while 1 bp mismatch is not likely to be sufficient to prevent binding, differences in gene silencing activity may be sufficient, in some cases, to enable the development of peptide-PNAs that would be selectively antibacterial to *S*. Typhimurium. Three separate BLAST analyses were done: 1) TIR sequences of genes described as essential in *S*. Typhimurium against the TIRs of *E. coli* essential genes; 2) TIRs of *S*. Typhimurium genes described as essential in *E. coli*, against TIRs of *E. coli* essential genes and 3) the TIRs of *E. coli* essential genes against TIR sequences of genes described as essential in *S*. Typhimurium (Tables S2–4 in Information S1). Using the essential genes of *S*. Typhimurium from the DEG database, and the −5 to +5 TIR region we identified 113 genes that could serve as targets for peptide-PNAs that would be selectively antibacterial to *S*. Typhimurium over *E. coli* (Table S2 in Information S1). Of these, 68 genes had orthologues not identified as essential in *E. coli*, 34 did not have orthologues in *E. coli* and of the 11 genes that had essential orthologues in *E. coli*, 5 of these had TIR sequences with >2 bp mismatches. The fact that peptide-PNA Se0001, was selectively antibacterial for *S*. Typhimurium, and designed to target a gene not reported to be essential in *S*. Typhimurium indicates that orthologues of genes that are identified as essential in *E. coli* are likely to be essential in *S*. Typhimurium. Furthermore, *E. coli* genes have been identified as essential by failure to construct a specific knockout, whereas those of *S*. Typhimurium were identified by trapping lethal insertions [19].

**Figure 3:**
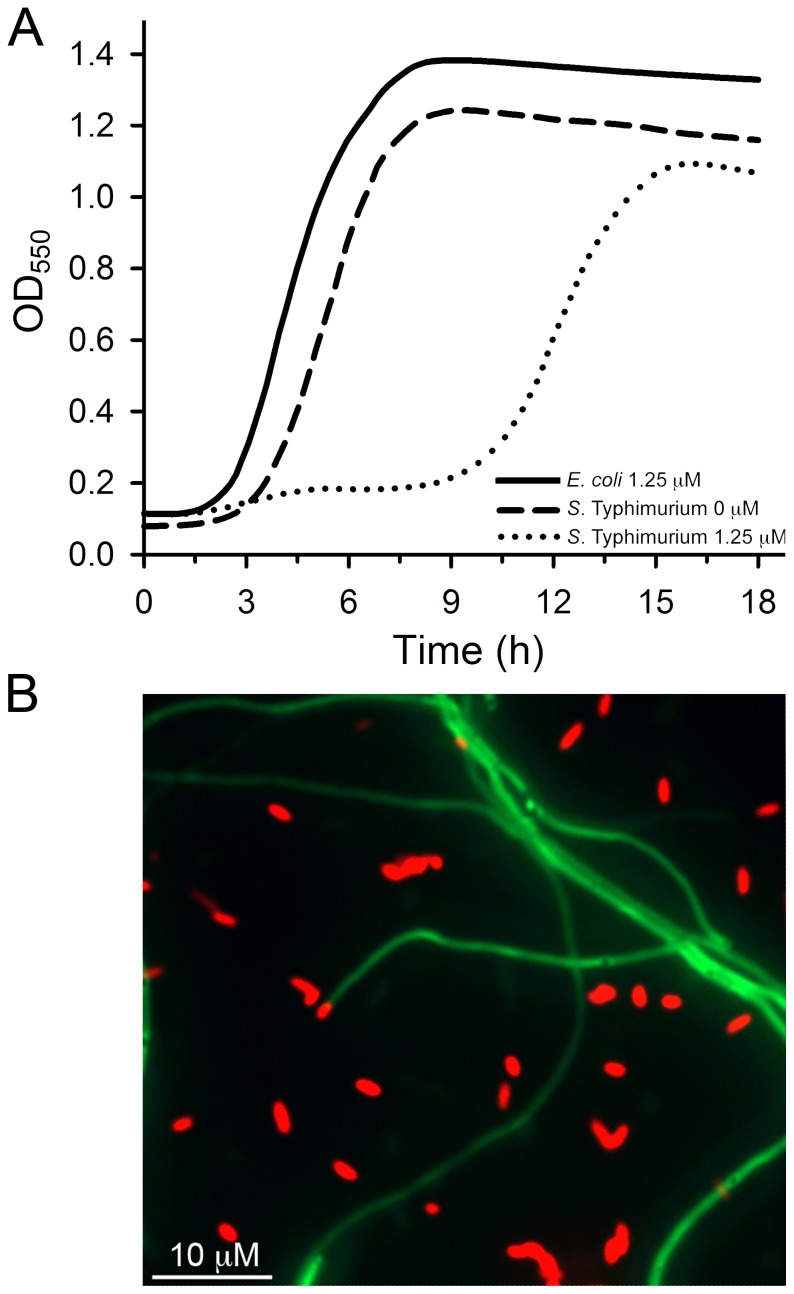
*S*. Typhimurium-selective growth inhibition. Peptide-PNA Se0002 was designed to target the −5 to +5 region of the translational initiation region (TIR) of *ftsZ* in *S*. Typhimurium. Se0002 has 2 base pair mismatches in the TIR of *ftsZ* in *E. coli*. (A) Growth curve analysis of Se0002 in pure culture. *E. coli* growth in the presence of 1.25 µM Se0002 (solid line) was identical to that of untreated controls (not shown). *S*. Typhimurium growth was inhibited in the presence of 1.25 µM of Se0002 (dotted line) relative to the untreated control (dashed line). Growth in the treated samples after 10 hrs was not due to resistance (see text for details). (B) Mixed cultures of GFP-labeled *S*. Typhimurium AC02 and DsRed-labeled *E. coli* AC01 were treated with 1.25 µM Se0002; and imaged by fluorescence microscopy after 6 hrs of incubation. The filamentous growth phenotype was only observed in *S*. Typhimurium AC02 and is consistent with silencing of *ftsZ* expression.

We applied the bioinformatic screening technique described above to identify peptide-PNAs that would discriminate between *S*. Typhimurium and *E. coli* using the essential genes of *E. coli* as the query sequences. This analysis identified a further 93 orthologous genes that could potentially act as targets for discriminatory peptide-PNAs (Table S3 in Information S1). Of these, 47 genes had TIRs with >2 bp mismatches between the two species, from which *ftsZ* was chosen for further study. This target was chosen for the reasons given above, and because gene silencing of *ftsZ* should result in a cell filamentation phenotype that would enable microscopic evaluation of the specificity of the peptide-PNA in mixed culture. DsRed-labelled *E. coli* AC01, and GFP-labelled *S*. Typhimurium AC02 were exposed to peptide-PNA Se0002 at concentrations ≤5 µM. *E. coli* AC01 was unaffected by Se0002 at all concentrations tested, whereas *S*. Typhimurium growth was inhibited at 1.25 µM (0.5×MIC), with a lag phase ca. 7.5 hrs longer than that of the untreated sample (Figure 3A). Growth of *S*. Typhimurium observed in Se0002-treated cultures after 10. 5 hrs was not due to the generation of spontaneous resistance mutants, as samples of cells taken after 14 hrs of incubation, passaged into fresh media containing the same concentration of Se0002, exhibited identical growth kinetics to the parent culture. Growth is more likely due to the effective concentration of Se0002 falling below that required to inhibit translation of *fts*Z; caused by the peptide-PNA accumulating in non-growing cells, adsorbing to the plastic of the well, or proteolysis of the carrier peptide. Mixed cultures of *E. coli* AC01 and *S*. Typhimurium AC02 prepared as above, treated with 1.25 µM of Se0002 were sampled after 6 hrs of growth and were observed by fluorescence microscopy (Figure 3B). As predicted, *E. coli* AC01 treated with Se0002 had an identical phenotype to untreated controls, whereas *S*. Typhimurium AC02 cells displayed a distinct filamentous phenotype only upon treatment with Se0002. This phenotype is consistent with previous studies using anti-*fts*Z peptide-PNAs in *E. coli* [17]. These results, and those of the mixed culture experiments detailed above, prove that it is possible to employ antisense-based molecules as species-selective antimicrobial agents in mixed culture.

## Discussion

The aim of this study was to test the hypothesis that peptide-PNAs can be applied as species-selective antimicrobial compounds. Using mixed cultures, we demonstrate the feasibility of using PNAs as species-selective antisense antibacterials. Antibacterial peptide-PNAs evaluated against *B. subtilis*, *E. coli*, *K. pneumoniae* and *S.* Typhimurium, both in single and mixed cultures, with the exception of Ec1000, displayed detectable antibacterial activity against the intended species only. Peptide-PNA treatment of mixed cultures enabled selective growth inhibition that, in theory, could not be achieved using the twenty antibiotics evaluated in this study. Our findings suggest that PNAs are candidates for narrow-spectrum antimicrobials. For species-selectivity, 16S rRNA would appear to be a logical candidate. However, while peptide-PNAs targeted to 16S rRNA have been demonstrated to be bactericidal and sequence selective [11], [20], prior to this study, species selectivity has not been observed and may be difficult to achieve due to sequence conservation within functional regions of 16S rRNA.

The observation that En108 has different activity in three closely related species might be explained by binding of the PNA to the TIRs of 1-base mismatched off-target essential genes simultaneously in *K. pneumoniae* and *S.* Typhimurium, and only the intended target in *E. coli*. Antisense-based antimicrobials have been demonstrated to show greater growth inhibitory activity when the expression of multiple essential genes are simultaneously inhibited [9]. This may also explain the weak synergistic effect of Kp0001 and Se0001 when used in combination in cultures of *B. subtilis*. In addition, there may be mechanistic differences in the gene silencing itself (binding efficacy, *T*_m_ differences caused by mismatches, and location of the off-target relative to TIR) or differences in the stringency of requirement for the target and off-target genes, i.e. a small reduction in the mRNA pool of the two off-targets in *K. pneumoniae* may be better tolerated than that of the off-targets in *S.* Typhimurium [17]. Differences in the susceptibility of the three species to En108 may also be explained by uptake efficiency; the transporter protein SbmA has recently been identified as required for PNA uptake in *E. coli* [17], [21]. The authors show that the peptide-PNA crosses the outer membrane, followed by protease degradation of the peptide carrier in the periplasmic space, and SbmA-mediated transport of the free PNA across the inner membrane. Degradation of the peptide carrier, may account for the slight increase in growth observed in non-target species. It is interesting that the *E. coli sbm*A gene shares 86% and 92% similarity to its orthologues in *K. pneumoniae* and *S*. Typhimurium respectively; determining if SbmA orthologues have different PNA uptake kinetics may aid predicting the likely susceptibility of species to PNAs. *B. subtilis* is not reported to have a homologue of SbmA. However, it is sensitive to bleomycin, a glycopeptide transported by SbmA into the cytoplasm of *E. coli*. Homology searching of the *B. subtilis* genome using the SbmA sequence from *E. coli*, reveals a number of ABC-type transporters, such as YgaD (Figure S2 in Information S1) that may perform a similar function as SbmA. Elucidation of the mechanism of PNA uptake in Gram-positive species will aid in their development as antibacterial compounds.

The finding that PNA Ec1000, designed to silence the *mur*A gene of *E. coli*, was antibacterial to *S.* Typhimurium was unexpected. The parameters described for the design of species-selective PNAs theoretically selects for PNAs to the TIRs of essential genes in only the target species. An analysis of the binding sites (allowing for 1 bp mismatch) for Ec1000 in *S.* Typhimurium shows that it binds intragenically to a number of essential genes (*ala*S, *fus*A, *hem*E and *rpl*F) that are reported to be toxic upon overproduction [22]; peptide-PNAs can significantly elevate gene expression when binding downstream of the TIR [18]. It is also possible that the Ec1000 PNA may bind to a non-coding RNA, which can act as regulatory elements [23]. While we could not identify any potential binding between Ec1000 and known non-coding RNAs in *S.* Typhimurium [24], there may be as yet unidentified non-coding RNAs that are transcribed from, or interact with, intergenic Ec1000 binding sites; disruption of a RNA antitoxin is one possible mechanism that would lead to bactericide [25]. The elucidation of the mechanism responsible for Ec1000-induced growth inhibition in *S.* Typhimurium is required for the continued development of species-selective antibacterial PNAs; understanding of the mechanism will enable the design parameters of peptide-PNAs to be modified to exclude likely off-target effects and/or identification of new targets for gene-silencing antimicrobials.

Comparative genomics between sensitive and resistant species, qRT-PCR [17] and the introduction of point mutations within putative targets could be applied to identify the cause of the off-target selectivity in *S.* Typhimurium. While the unexpected activity of Ec1000 prevented its use as an *E. coli* species-selective peptide-PNA, our *in-silico* analysis, and the identification of *E. coli*-specific TIRs (Table S4 in Information S1) suggest that finding targets that are amenable for discriminating *E. coli* from closely related species is readily achievable. There are 46 targets, in the −5 to +5 TIR region of essential genes in *E. coli*, that have >2 bp mismatches with orthologs in *S*. Typhimurium (Table S3 in Information S1). The *in silico* approach was applied in the successful design of Se0002, and thus a peptide-PNA raised to the same target in *E. coli*, will likely discriminate *E. coli* for *S*. Typhimurium. Nevertheless, the possibility for cross-reactivity to occur in bacterial species that are part of the microbiome cannot be excluded, as microbiomes are dynamic and unique to host species. The availability of in-depth microbiome sequencing will aid in the design of species-selective PNAs through exhaustive prediction of binding sites in all species of the microbiome.

Relative to currently used antibiotics, PNAs provide new opportunities for the design of ultra-narrow-spectrum antimicrobials, where the primary target is dictated by the nucleic acid sequence. Of course, perfectly specific pathogen targeting may never be possible, but the results presented here show clearly that large improvements are possible over currently used drugs, thus enabling the reduction of antibiotic resistance and secondary infection, through the reduction of off-target antibiosis. Finally, while the mode of action of peptide-PNA antimicrobials is well understood [11], [15], [26], [27], differences in uptake of the peptide-PNA, species sensitivity and the effect of non-target binding remain important areas for future experimentation.

## Materials and Methods

### Bacterial strains and growth conditions

A list of the strains used in this study is given in Table 3. All strains were grown in Miller's modified Luria broth (MMLB; Sigma-Aldrich, UK) with constant shaking (200 rpm) at 37°C. For mixed-culture growth, MMLB was inoculated with 1×10^4^ CFU/ml; the proportion of each species needed to give reproducible species counts after 16 h of growth at 35°C was experimentally determined (Table S5 in Information S1).

**Table 3:** Bacterial strains used in this study.

Strain	Source	Genotype	Characteristic
*Bacillus subtilis* subsp. *subtilis* 168	ATCC^a^ 23857	*trpC2*	Genome sequenced strain
*Escherichia coli* DH10B	Invitrogen	*F^−^ endA1, recA1, galE15, galK16, nupG, rpsL,* Δ*lacX74, Φ80lacZ*Δ*M15, araD139,* Δ*(ara,leu)7697, mcrA,* Δ*(mrr-hsdRMS-mcrBC), λ^−^*	Genome sequenced strain, parent of *E. coli* AC01
*E. coli* AC01	This study	As above, pDsRed-Express2	Expression of DsRed fluorescent protein
*Klebsiella pneumoniae* subsp. *pneumoniae*	ATCC 700721	n/a	Genome sequenced strain
*Salmonella enterica* serovar Typhimurium LT2	SGSC^b^ 1412	n/a	Genome sequenced strain
*S.* Typhimurium LT2 substr JR501	SGSC 1593	*hsdSA29, hsdSB121, hsdL6, metA22, metE55,1 trpC2, ilv-452, H1-b, H2-e,n,x* (cured of Fels 2), *fla-66, nml, rpsL120, xyl-404, galE719*	Restriction-deficient, modification-proficient cloning strain of *S*. Typhimurium LT2, parent of *S*. Typhimurium AC02
*S.* Typhimurium AC02	This study	As above, pGFPuv	Expression of green fluorescent protein

a American Type Culture Collection.

b *Salmonella* Genetic Stock Center.

### Design of species-specific peptide PNAs

General guidelines for the design of antibacterial peptide-PNAs are described elsewhere [11], [15], [26], [27]. Criteria used for the design of species-specific peptide-PNAs are described in the results section. The Database of Essential Genes [28] and BLAST [29] were used to identify any essential gene homologues present in all four species used in this study. The Artemis program [30] was used to extract twenty base-pairs (−10 to +10 bases relative to the start codon) of the TIRs from the genome sequences of *Bacillus subtilis* [31], *Escherichia coli* DH10B [32], *Klebsiella pneumoniae* and *Salmonella enterica* serovar Typhimurium LT2 [19] (GenBank accession numbers: AL009126; CP000948; CP000647; and AL513382 respectively). The 20 bp TIRs from gene homologues were aligned in Clustal X version 2.0 [33] and the number of base-pair mismatches between species was determined. The predicted thermal stability (*T*_m_) of PNA/DNA duplexes was determined according to formula of Giesen et al. 1998 [34]. A genomic analysis of the possible binding sites of the PNAs within their target species was conducted in Artemis, using a cut-off of greater than 2 bp mismatches. Secondly, to comprehensively examine the number of potential antibacterial PNAs that could be used to discriminate between two closely related species, a semi-automated method was employed: genome sequences were used to identify the start codon positions of essential genes from *E. coli* DH10B and *S.* Typhimurium LT2. A custom PERL script was used to extract the −5 to +5 bases relative to the start codon of each gene. The 10 bp sequences were used for an all-against-all comparison using standalone BLAST [29], [35] to identify the TIRs of essential genes that were amenable to the design of species specific PNAs. The peptide-PNAs used in this study and their properties are listed in Table 1.

### Antimicrobial susceptibility and peptide-PNA minimal inhibitory concentration (MIC) testing

Strains were tested with twenty different antibiotic disks (Oxoid, UK) representing the major classes of antimicrobial compounds. Tests were done according to the standardized disc susceptibility testing method of the British Society for Antimicrobial Chemotherapy [36], [37]. The minimum inhibitory concentration (MIC) of the peptide-PNA conjugants were determined using a method modified from Hacek et al [38] and Friedman et al [39]: Peptide-PNA conjugants, obtained as lyophilized powder (Panagene, Korea), were dissolved in ddH_2_0. MIC assays were performed in an ultra low-bind (Costar, UK) polystyrene 96-well plate format in a final volume of 150 µl MMLB. An extended gradient of peptide-PNA concentrations was created by combining five sets of twofold serial dilutions from four starting concentrations (10, 4.8, 3.2 and 3 and 2 µM); giving 55 final peptide-PNA concentrations which extended over five rows of the 96-well plate. All cultures were incubated at 35°C for 16 h without shaking or agitation. Each peptide-PNA MIC calculation was performed in triplicate, each replicate representing a different starting colony. Mixed-culture experiments were conducted as above with a 1×10^4^ CFU/ml starting inoculum. For growth curve analysis, 200 µl cultures were grown in a BioTek PowerWave HT spectrophotometer, under constant agitation at 37°C in a 96-well plate covered with a breathable film. Growth (OD_550_) was monitored every 5 mins, each experiment was performed in triplicate.

### Microscopy

Cells prepared for fluorescence imaging were grown in a BioTek PowerWave HT spectrophotometer as above. After six hours 10 µl of culture was removed, washed and resuspended in 1 X PBS by centrifugation (13, 000×*g*). Cells were applied to an agarose pad [40] and viewed using an epifluorescence Leica DMRB microscope. An EXi Aqua CCD camera (QImaging) and Image Pro Plus (MediaCybernetics) were used for image acquisition and processing.

### Species identification

In mixed-culture experiments, colonies on MMLB plates were identified using a combination of phenotypic and genotypic properties. In order to rapidly identify species post peptide-PNA treatment, we designed a species-specific PCR-based identification method. Primer sets for peptide deformylase (*def*) were designed that yielded different sized amplicons for each species: *B. subtilis* (352 bp), *E. coli* (394 bp), *K. pneumoniae* (231 bp) and *S*. Typhimurium (280 bp) (see Table S6 in Information S1). All colonies were picked from the plates with the most countable dilution (30–50 cfu per plate) and used directly for colony multiplex PCR in 10 µl reaction volumes according to the simplified method of Menossi et al. [41]. Colonies were identified using standards prepared from pure cultures. Furthermore, in the three-species mixed-culture experiments, colonies were identified using the colony PCR protocol with 16S rRNA gene primers. The sequences of both strands of the resulting amplicons were determined with the BigDye (version 3.1) cycle sequencing kit and a 3730 DNA analyzer (Applied Biosystems, UK). Species identity was confirmed using the SEQMATCH function of the Ribosomal Database Project [42]

## Supporting Information

Information S1 Figure S1. Venn diagram of the fifteen possible species combinations (lettered A–O) in a theoretical bacterial community composed of the four species used in this study. The key on the right indicates the antimicrobial agent that would result in the bactericide of the desired species target(s). Peptide-PNAs are in bold. Only the peptide-PNAs designed in this study are capable of species-specific bactericide for individual (C and D) and mixed (M) Gram-negative species. * Peptide-PNA (Ec1000) was non-specific, but a number of *E. coli*- specific PNAs were designed (Table S4–6 in Information S1) that have yet to be evaluated. Figure S2. The predicted structures of the *E. coli* PNA transporter protein SbmA (pink) and YgaD (blue) of *B. subtilis*. Structures were predicted using the I-TASSER platform [1]. YgaD was identified by homology searching using HHPred [2] with SbmA as the input sequence. Protein sequences were aligned and rendered by PyMOL [3]. Table S1. Antibiotic susceptibility of strains used in this study. Table S2. *S. Typhimurium*-specific PNAs. Table S3. *S. Typhimurium*-specific PNAs designed using essential genes from *E. coli.* Table S4. *E. coli*-specific PNAs. Table S5. Number of cells used for inoculation of mixed culture. Table S6. Oligonucleotides used in this study.
(DOCX)
