# Supplementary material for: Species-Selective Killing of Bacteria by Antimicrobial Peptide-PNAs
Source: PLoS One. 2014 Feb 18;9(2):e89082. doi: 10.1371/journal.pone.0089082 (PMC3928365; doi:10.1371/journal.pone.0089082)
Supplement: Information S1 — Figure S1. Venn diagram of the fifteen possible species combinations (lettered A–O) in a theoretical bacterial community composed of the four species used in this study. The key on the right indicates the antimicrobial agent that would result in the bactericide of the desired species target(s). Peptide-PNAs are in bold. Only the peptide-PNAs designed in this study are capable of species-specific bactericide for individual (C and D) and mixed (M) Gram-negative species. * Peptide-PNA (Ec1000) was non-specific, but a number of E. coli- specific PNAs were designed (Table S4–6 in Information S1) that have yet to be evaluated. Figure S2. The predicted structures of the E. coli PNA transporter protein SbmA (pink) and YgaD (blue) of B. subtilis. Structures were predicted using the I-TASSER platform [1]. YgaD was identified by homology searching using HHPred [2] with SbmA as the input sequence. Protein sequences were aligned and rendered by PyMOL [3]. Table S1. Antibiotic susceptibility of strains used in this study. Table S2. S. Typhimurium-specific PNAs. Table S3. S. Typhimurium-specific PNAs designed using essential genes from E. coli. Table S4. E. coli-specific PNAs. Table S5. Number of cells used for inoculation of mixed culture. Table S6. Oligonucleotides used in this study. (DOCX) [file pone.0089082.s001.docx]

**Species-selective killing of bacteria by antimicrobial peptide-PNAs**

Madhav Mondhe^1^, Ashley Chessher^1^, Shan Goh^2^, Liam Good^2^ and James E. M. Stach^1^

*School of Biology, Newcastle University, Newcastle upon Tyne, United Kingdom^1^, Department of Pathology and Infectious Diseases, Royal Veterinary College, University of London, London, United Kingdom^2^*

**Supporting Information**

**Figs S1 and S2**

**Tables S1-S6**


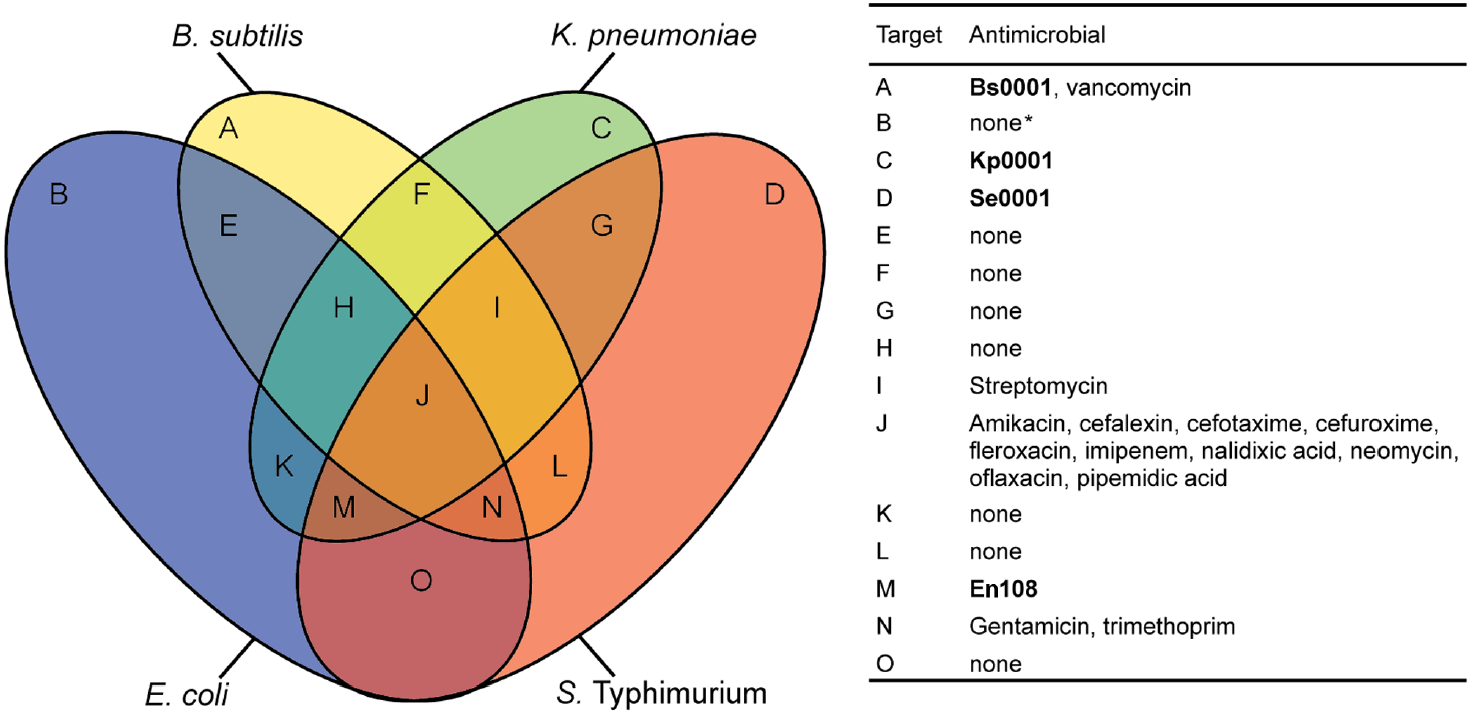


**Figure S1.** Venn diagram of the fifteen possible species combinations (lettered A-O) in a theoretical bacterial community composed of the four species used in this study. The key on the right indicates the antimicrobial agent that would result in the bactericide of the desired species target(s). Peptide-PNAs are in bold. Only the peptide-PNAs designed in this study are capable of species-specific bactericide for individual (C and D) and mixed (M) Gram-negative species. * Peptide-PNA (Ec1000) was non-specific, but a number of *E. coli*- specific PNAs were designed (Table S4) that have yet to be evaluated.


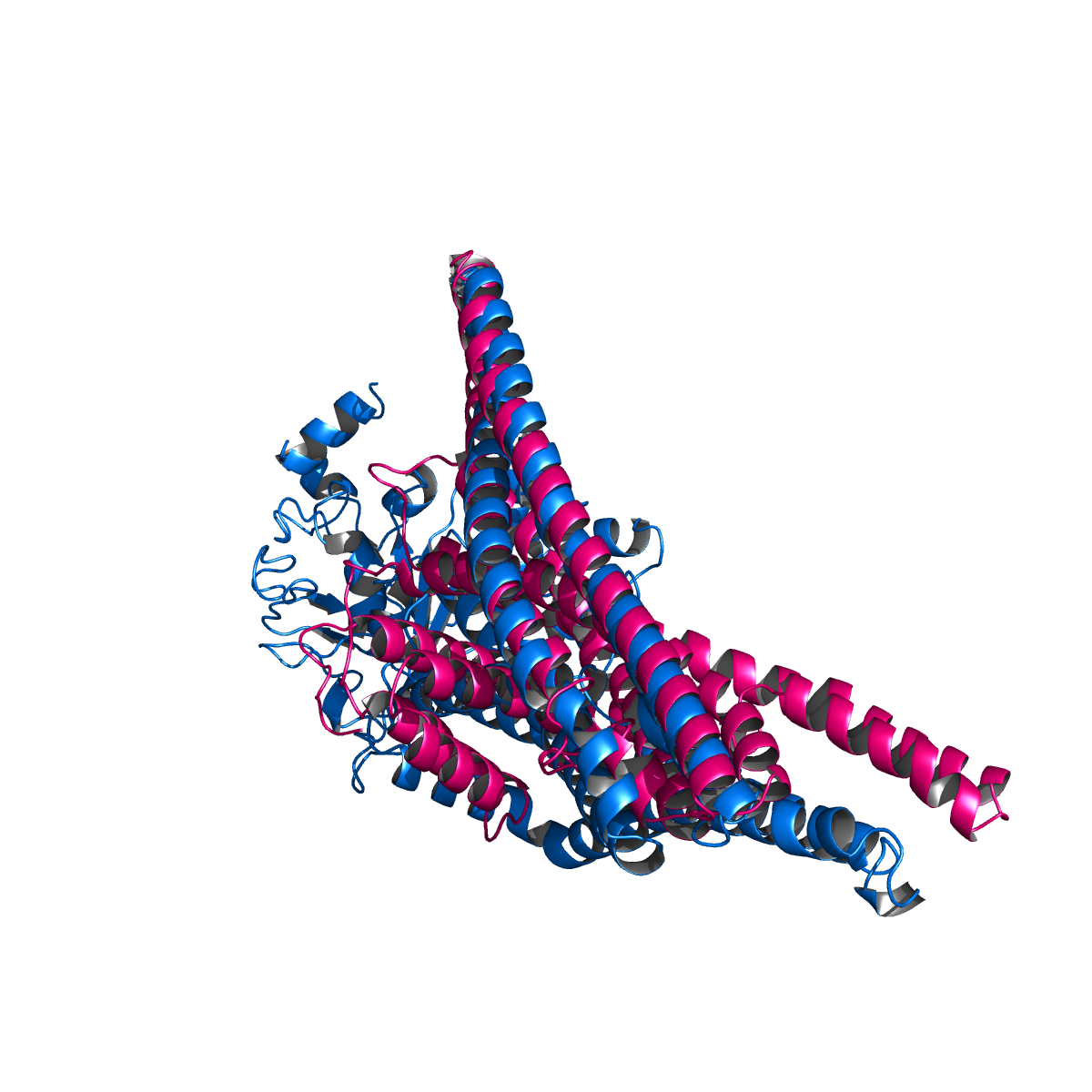


**Figure S2.** The predicted structures of the *E. coli* PNA transporter protein SbmA (pink) and YgaD (blue) of *B. subtilis*. Structures were predicted using the I-TASSER platform [1]. YgaD was identified by homology searching using HHPred [2] with SbmA as the input sequence. Protein sequences were aligned and rendered by PyMOL [3]

| Target | Class | Antibiotic | Amount per disc (μg) | Sensitive (S)/Resistant (R) | | | |
| --- | --- | --- | --- | --- | --- | --- | --- |
|  |  |  |  | *B*. *subtilis* | *E. coli* | *K*. *pneumoniae* | *S*. Typhimurium |
| Cell wall synthesis | Penicillins | Methicillin | 10 | S | S | R | S |
|  |  | Amoxicillin | 25 | S | S | R | S |
|  | Cephalosporins | Cefalexin | 30 | S | S | S | S |
|  |  | Cefuroxime | 30 | S | S | S | S |
|  |  | Cefotaxime | 30 | S | S | S | S |
|  | Glycopeptide | Vancomycin | 30 | S | R | R | R |
|  | Carbapenem | Imipenem | 10 | S | S | S | S |
| Ribosome 30S | Aminoglycosides | Amikacin | 30 | S | S | S | S |
|  |  | Streptomycin | 30 | S | R | S | S |
|  |  | Neomycin | 30 | S | S | S | S |
|  |  | Gentamicin | 30 | S | S | R | S |
| Ribosome 50S | Macrolides | Erythromycin | 15 | S | R | R | R |
|  |  | Azithromycin | 15 | S | R | R | R |
|  | Lincosamides | Lincomycin | 15 | S | R | R | R |
|  |  | Clindamycin | 10 | S | R | R | R |
| Nucleic acid synthesis | Quinolones | Nalidixic acid | 30 | S | S | S | S |
|  |  | Pipemidic acid | 20 | S | S | S | S |
|  | Fluoroquinolones | Fleroxacin | 5 | S | S | S | S |
|  |  | Oflaxacin | 5 | S | S | S | S |
| Tetrahydrofolic acid synthesis |  | Trimethoprim | 5 | S | S | R | S |

**Table S1. Antibiotic susceptibility of strains used in this study**

**Table S2.** *S.* Typhimurium-specific PNAs

| Gene | PNA sequence | | | *T*_m_ | Orthologue essential in *E. coli* | No. of mismatches with *E. coli* orthologue | Off-target essential gene |
| --- | --- | --- | --- | --- | --- | --- | --- |
| *acrB* | | GGCATGTCTT | 55.8 | | no | 0 | no |
| *adhE* | | GCCATAATGC | 56.4 | | no | 0 | no |
| *adk* | | CGCATTACGA | 59.2 | | yes | 1 | no |
| *avrA* | | ATCATCTTTA | 40.5 | | n/a | n/a | no |
| *bcr* | | GTCACATCGA | 58.6 | | no | 1 | no |
| *cheA* | | CTCACGCTAT | 51.1 | | no | 1 | no |
| *clpB* | | CGCATAACTC | 53.2 | | no | 0 | no |
| *clpX* | | GTCATGAGTC | 55.1 | | no | 1 | no |
| *csdA* | | TTCATGATAT | 42.8 | | yes | 2 | no |
| *cstA* | | TTCATAGTTG | 46.3 | | no | 0 | no |
| *dcoC* | | TTCATAATAA | 42.5 | | n/a | n/a | *yjgM^c^* |
| *ddl* | | GCCATGTTGC | 59.3 | | no (*ddlB)* | 2 | no |
| *dfp* | | CTCATCATGT | 46.8 | | yes | 2 | *lpdA^c^, rimM^c^* |
| *dnaK* | | CCCATCTAAA | 50.1 | | no | 0 | no |
| *fic* | | CTCATAGTGC | 51.2 | | no | 1 | no |
| *ftsZ^a^* | | AACATAATCT | 46.1 | | yes | 2 | no |
| *gcd* | | GCCATAAAAA | 57.4 | | no | 1 | no |
| *glmM* | | CTCATAGCGT | 53.8 | | no | 0 | no |
| *glnA* | | GACATACTTG | 50.5 | | no | 1 | no |
| *glyS* | | GACATGGCCG | 68.2 | | yes | 1 | no |
| *hisI* | | AACATATCCT | 46.7 | | no | 0 | no |
| *hisM* | | ATCACAAATC | 50.4 | | no | 2 | no |
| *hrpA* | | AGCATCAGTG | 59.9 | | no | 6 | no |
| *hybB* | | GATCGTCTCC | 54.2 | | no | 0 | no |
| *icdA* | | TCCATTCACC | 50.5 | | no | 0 | no |
| *katG* | | CTCATATCTC | 39.4 | | no | 4 | no |
| *lpxA* | | ATCACGAATC | 53.7 | | yes | 1 | no |
| *lysS* | | GACATGTTGA | 57.4 | | no | 1 | no |
| *maf* | | GGCATATTTT | 47.9 | | n/a | n/a | no |
| *metG* | | GTCATAGTGG | 56.7 | | yes | 1 | *rho* |
| *miaE* | | GTCATACTAT | 43.0 | | n/a | n/a | no |
| *mig-3* | | TACATGGTGT | 55.1 | | n/a | n/a | no |
| *mrp* | | TTCATCCCGA | 55.8 | | no | 0 | no |
| *murB* | | GTCATGGCTG | 60.2 | | yes | 2 | *nrfC^c^* |
| *murG* | | CTCATCGAGA | 56.7 | | yes | 1 | no |
| *mviN* | | TGCATGGCTG | 63.0 | | no (*mur*J)*^b^* | 3 | no |
| *napH* | | GCCATCTTTC | 48.7 | | no | 2 | no |
| *nrfC* | | CTCATGGCTG | 56.6 | | no | 0 | *murB^c,d^* |
| *pabB* | | ATCATTCCTG | 47.2 | | no | 5 | no |
| *pdxK* | | CCCATAAAAT | 49.5 | | no | 2 | no |
| *phoR* | | AGCACGCGTC | 67.5 | | no | 4 | no |
| *polA* | | ACCATAATGT | 50.7 | | no | 0 | no |
| *prfB* | | GTTATTCACC | 46.5 | | no | 8 | no |
| *pstS* | | TTCATAATGT | 44.5 | | no | 0 | no |
| *pyrF* | | GTCATGACCA | 58.2 | | no | 0 | *hflK^c^,* STM2913*^c^*, STM4259*^c^* |
| *recC* | | AACATAATTA | 45.5 | | no | 4 | no |
| *recF* | | GACATATATT | 42.4 | | no | 5 | no |
| *rep* | | CGCATGGGAA | 69.4 | | no | 3 | no |
| *rfaB* | | TTCATATATA | 36.1 | | no | 2 | *trxA^c^* |
| *rfaL* | | AGCATCTTTT | 47.2 | | no | 1 | no |
| *rfbK* | | TTCATTAGTC | 43.5 | | no (*cps*G) | 5 | no |
| *rfbV* | | AGCATATGAT | 51.8 | | n/a | n/a | no |
| *rhsE* | | TGCACGTTCT | 56.1 | | no | 8 | no |
| *rnb* | | AACATATTCG | 50.0 | | no | 3 | no |
| *rnt* | | GACATCTGAG | 56.8 | | no | 1 | no |
| *rplC* | | ATCATTGTAT | 43.0 | | yes | 1 | no |
| *rpmJ* | | TGCATTTCAT | 47.9 | | no | 3 | no |
| *rpoD* | | TCCATAAGAC | 52.3 | | no | 0 | no |
| *ruvB* | | TCCAGCACCG | 64.8 | | no | 0 | no |
| *sbcB* | | GTCACTTGAT | 50.9 | | no | 4 | no |
| *sinI* | | TGCATTCAGA | 56.5 | | n/a | n/a | no |
| *smvA* | | AACTCTCCCT | 50.5 | | n/a | n/a | no |
| *sopA* | | TTCATTAGAA | 47.4 | | n/a | n/a | no |
| *sspB* | | TCCATACAGG | 56.0 | | no | 1 | no |
| STM0268 | | TCCATGAATA | 50.4 | | n/a | n/a | no |
| STM0345 | | TGCATTTCGG | 57.6 | | n/a | n/a | no |
| STM0438 | | TCCACAGATA | 53.9 | | n/a | n/a | no |
| STM0557 | | CTCATTCTTA | 39.5 | | n/a | n/a | no |
| STM0858 | | TTCATTATTA | 37.1 | | n/a | n/a | no |
| STM1003 | | AACATAATGA | 52.7 | | n/a | n/a | no |
| STM1008 | | TCCATGATTT | 46.8 | | n/a | n/a | STM2633*^c^* |
| STM1097 | | TTCATGCTTC | 46.8 | | n/a | n/a | *yfiF^c^* |
| STM1128 | | ATCATAGTTC | 44.7 | | n/a | n/a | no |
| STM1129 | | GACATCCAGT | 57.2 | | n/a | n/a | no |
| STM1133 | | ATCATTTATC | 38.7 | | n/a | n/a | no |
| STM1552 | | TTCACAACTT | 48.1 | | n/a | n/a | no |
| STM1556 | | CTCATAATGG | 49.3 | | n/a | n/a | *tnaB^c,d^* |
| STM1559 | | ATCATGATGC | 53.4 | | n/a | n/a | no |
| STM1560 | | CTCATTTGTC | 44.0 | | n/a | n/a | no |
| STM1637 | | GCCATAATAT | 47.4 | | n/a | n/a | no |
| STM2273 | | TTCATAATTT | 39.0 | | n/a | n/a | *rplS^c,d^,* STM0857*^c^* |
| STM2274 | | CTCATTTTGC | 45.4 | | n/a | n/a | no |
| STM2406 | | ATCATGACCT | 51.6 | | n/a | n/a | STM2913*^c^* |
| STM2619 | | GCCATGCTCA | 60.1 | | n/a | n/a | no |
| STM2633 | | TCCATGATTT | 46.8 | | n/a | n/a | STM1008*^c^* |
| STM2844 | | AACACAAATT | 53.3 | | n/a | n/a | no |
| STM2913 | | GTCATGACCT | 54.5 | | n/a | n/a | *hlfK^c^*, *pyrF^c^,*  STM2406*^c^* |
| STM3012 | | ATCATGCTAC | 49.3 | | n/a | n/a | no |
| STM4262 | | TCCATAAGTA | 49.0 | | n/a | n/a | no |
| *tdk* | | GCCATTGGTC | 58.7 | | no | 3 | no |
| *thrS* | | GGCATGTTAT | 53.6 | | no | 1 | no |
| *topB* | | CGCATCAGGT | 62.6 | | no | 4 | no |
| *trmD* | | AACACAAGCC | 62.4 | | yes | 5 | no |
| *trxA* | | CTCATATATA | 36.1 | | no | 0 | *rfaB^c^* |
| *ulaA* | | TCCATAAGCG | 57.9 | | no | 1 | no |
| *uppS* | | AACATAACGC | 58.2 | | no (*isp*U) | 1 | no |
| *vacB* | | GACATTGAGG | 61.1 | | no (*rnr*) | 3 | no |
| *wcaD* | | ACTCCTCCAG | 55.0 | | no | 0 | no |
| *yafD* | | CGCATCGCAT | 60.8 | | no | 6 | no |
| *yaiZ* | | CGCATAACGA | 61.9 | | no | 6 | no |
| *ybeX* | | TCATGGCGTC | 60.6 | | no | 0 | no |
| *ybjO* | | CCCAATGAAT | 54.3 | | no | 5 | no |
| *ycaL* | | TTCATACTAA | 43.0 | | no | 3 | no |
| *yccJ* | | GGCATTCGAA | 63.0 | | no | 2 | no |
| *ychK* | | CTCATCGTAC | 49.1 | | no (*rss*A) | 3 | no |
| *ychM* | | ATCACATGTG | 53.4 | | no | 5 | no |
| *yciG* | | GCCATATTAT | 44.7 | | no | 1 | no |
| *ydcN* | | TCCATTTTCC | 44.6 | | no | 4 | no |
| *ydgP* | | AGCATGGTTT | 57.1 | | no (*rsx*G) | 4 | no |
| *ydiN* | | GACATTTGTT | 49.1 | | no | 3 | no |
| *yeaZ* | | CGCATGAGGT | 65.3 | | no | 3 | no |
| *yebW* | | AACATTACAT | 47.4 | | no | 1 | no |
| *yecS* | | TGCATTCGCG | 61.8 | | no | 4 | no |
| *yeeF* | | GTCATTCTCC | 47.4 | | no | 4 | no |
| *yfaW* | | TCCATTTTAA | 43.5 | | no (*rhm*D) | 6 | no |
| *yfgE* | | GACATAAAAT | 51.3 | | no (*hda*) | 7 | no |
| *yhcK* | | TCCATTCATG | 48.3 | | no (*nan*R) | 4 | no |
| *yjcD* | | GACATAGAAA | 57.1 | | no | 2 | no |
| *yjjY* | | GTCATGTTAC | 48.9 | | no | 0 | no |
| *yliB* | | GTCATGTTGT | 52.0 | | no (*gsi*B) | 3 | no |
| *yneI* | | GTCATGTTCT | 47.3 | | no (*sad*) | 5 | no |
| *yoaE* | | TCCATGACAA | 56.6 | | no | 0 | no |
| *ytfE* | | GCCATAGCCG | 64.2 | | no | 1 | no |
| *ytfP* | | CGCATTGCTA | 56.0 | | no | 0 | no |

*^a^* PNA used in this study.

*^b^* Difference in annotation of start codon location between *E. coli* K12 and *S.* Typhimurium. The start codon of *E. coli* was used.

*^c^* Off-target gene reported to be essential in *S*. Typhimurium with 9 or 10 bp matches with the PNA

*^d^* Off-target gene reported to be essential in *E. coli* with 9 or 10 bp matches with the PNA

**Table S3.** *S*. Typhimurium-specific PNAs designed using essential genes from *E. coli*

| Gene*^a^* | PNA sequence | *T*_m_ | No. of mismatches with *E. coli* orthologue | *O*ff-target essential gene |
| --- | --- | --- | --- | --- |
| *accC* | AACATGTTCG | 55.8 | 1 | no |
| *accD* | CTCATTGGTG | 53.0 | 2 | no |
| *asd* | TTCATAGCGT | 53.2 | 4 | no |
| *bcsB* (*yhjN*) | TTCATCGCAT | 50.6 | 1 | *ispB^e^* |
| *birA* | TTCATGCAAT | 50.6 | 1 | *mufS^e^* |
| *cca* | TTCACGCCAC | 58.9 | 1 | no |
| *cdsA* | AGCAAAAGCA | 66.3 | 1 | no |
| *coaD* | TGCATGACAG | 60.9 | 2 | no |
| *cysS* | AACATCGAAG | 60.2 | 1 | no |
| *def* | GACATAATTA | 46.7 | 2 | no |
| *dxs* | CTCATCGCAA | 56.3 | 4 | *ispB^e^* |
| *entD* | AGCATCGTTT | 54.8 | 5 | no |
| *era* | CTCATTCCAA | 48.6 | 1 | no |
| *fabA* | ACCATGTTTT | 48.3 | 1 | *der^e^* |
| *fabZ* | GTCAAAATAC | 51.5 | 1 | no |
| *fmt* | GACACGTTGG | 64.5 | 1 | no |
| *folA* | ATCATTAATT | 40.2 | 3 | no |
| *folC* | TTCATTGTTT | 42.2 | 3 | *folC^e^, pheT^d,e^, rplC^d,e^* |
| *folE* | GGCATTAATC | 52.0 | 2 | no |
| *folK* | GTCATACGGT | 57.5 | 1 | no |
| *ftsK* | CTCAAAAAAG | 55.0 | 1 | no |
| *ftsL* | ATCATGCGTT | 54.5 | 1 | no |
| *ftsQ* | GACATATCAG | 51.6 | 1 | no |
| *ftsX* | TTCATTGGCC | 54.5 | 2 | *lpxK^e^* |
| *groS* (*groES*) | CTCATTGATA | 44.6 | 1 | no |
| *hemC* | GTCATTATCA | 45.8 | 6 | no |
| *hemD* | CTCATGCGGG | 64.9 | 1 | no |
| *hemG* | TTCACGTGTT | 53.0 | 1 | no |
| *hemH* | CGCATTGCCG | 63.8 | 1 | no |
| *hemL* | CTCATGGAAA | 56.8 | 2 | no |
| *holA* | ATCATTAGTT | 43.4 | 1 | no |
| *holB* | TTCATGCCGC | 58.9 | 3 | no |
| *infC* | TTAATACTTT | 37.7 | 1 | no |
| *ispA* | TCCATTTACT | 42.7 | 4 | no |
| *ispE* | ATCATTTCAC | 50.7 | 6 | no |
| *ispF* | CGCATTATGC | 54.2 | 1 | no |
| *ispH* | TGCATGTTTA | 50.3 | 2 | no |
| *ligA* | TCCATATCAC | 46.5 | 1 | *ydfB^e^* |
| *lnt* | GCCATTTTTA | 46.5 | 4 | no |
| *lolB* | GTCATAGTGA | 54.6 | 1 | no |
| *lolC* (*ycfU*) | TAATGTACAT | 45.5 | 1 | no |
| *lptA* (*yhbN*) | TTCATAAGAG | 50.4 | 2 | no |
| *lptG* (*yjgQ*) | TGCATTATAC | 45.8 | 2 | *tadC^d,e^* |
| *lpxB* | GCCATTAACG | 57.3 | 1 | no |
| *map* | GCCATGAATT | 55.2 | 1 | no |
| *minD* | GCCATAAAAA | 57.4 | 1 | no |
| *minE* | GCCATAATTT | 48.0 | 1 | *ssb^e^* |
| *mraY* | AACATGAACC | 57.9 | 2 | no |
| *mreC* | TTCATAGGCT | 51.6 | 2 | *prfA^e^* |
| *mreD* | ACCATTACCC | 52.1 | 5 | no |
| *mukF* | CTCATAGGTT | 48.6 | 2 | *dnaN^e^* |
| *murA^b^* | TCCATTATTG | 43.5 | 4 | *hemK^d^, prmC^e^, valS^e^* |
| *murE* | GCCACCTGTT | 57.9 | 1 | no |
| *murF* | ATCATGCGAT | 55.0 | 1 | *rnc^e^* |
| *murI* | GCCATAAACT | 53.9 | 1 | no |
| *nadD* | TTCATATCAC | 43.1 | 1 | no |
| *nadE* | GTCATTTAAC | 46.0 | 1 | *hutH^d^*, STM0932*^d^* |
| *nrdA* | TTCATGTGGT | 54.0 | 3 | no |
| *nrdB* | GCCATTGAAG | 59.7 | 4 | no |
| *pgsA* | TGCATAGTGA | 57.6 | 1 | *lolB^e^* |
| *pheT* | TTCATTGTTT | 42.2 | 3 | *folC^e^, rplC^d,e^* |
| *plsC* | AGCATAACAA | 57.5 | 1 | *coaD^e^* |
| *pyrG* | GTCATGCGGA | 66.0 | 1 | *folK^e^* |
| *pth* | GCCACGTTTT | 55.9 | 2 | *hisS^d,e^* |
| *rho* | TTCATAATGG | 49.0 | 1 | *pstS^d^*, STM1556*^d^* |
| *ribA* | TGCATGTATT | 49.0 | 2 | no |
| *ribC* (*ribE*) | AACATATCTT | 43.4 | 3 | no |
| *ribD* | TGCATGGCTT | 58.6 | 1 | *rpsS^e^* |
| *ribE* (*ribH*) | TTCATATTCT | 37.8 | 1 | no |
| *rimN* (*yrdC*) | TTCACTTGTT | 45.4 | 3 | no |
| *rplU* | TACATAAAAA | 49.7 | 1 | no |
| *rpmA* | GCCATTTAAT | 47.9 | 3 | no |
| *rpsB* | GCCATGATTT | 52.5 | 1 | *murD^d,e^*, STM1008*^e^*, STM2633*^e^* |
| *rpsN* | TTCATTGATT | 43.5 | 4 | *groS^e^* |
| *rseP* (*yaeL*) | AGCATAAAAC | 56.1 | 2 | no |
| *secD* | AACACGGCAG | 69.1 | 1 | no |
| *ssb* | GCCATAATTG | 52.1 | 4 | *minE^e^* |
| *tadC* (*yfhC*) | GGCATTATAC | 50.4 | 6*^c^* | *lptG^e^* |
| *tdcF* (*yjgF*) | CTCATGATTT | 43.8 | 5 | *kdsB^d,e^*, *fabG^d,e^, yjeA^e^* |
| *tilS* | GTCATGATCC | 52.5 | 2 | no |
| *thrS* | GGCATGTTAT | 53.6 | 1 | no |
| *tmk* | CCCATTTTTT | 41.8 | 1 | no |
| *tnaB* (*mtr*) | GCCATTCAGC | 58.5 | 4 | no |
| *trpS* | GTCATTTTTT | 40.9 | 1 | *hemE^d^*, *narY^d^*, STM4259*^d^*, *ydiL^e^* |
| *ubiA* | TCCATCTTTA | 42.6 | 2 | no |
| *ubiB* | GTCATTTGGC | 55.2 | 1 | no |
| *waaU* (*rfaK*) | ATCATAATAG | 43.7 | 6 | no |
| *wzyE* (*wecF*) | CTCATTCGGC | 55.5 | 2 | no |
| *ydiL* | ATCATTTTTT | 38.1 | 4 | *narY^d^*, *ompN^d,e^*, STM4259*^d^*, *trpS^e^* |
| *yeaZ* | CGCATGAGGT | 65.3 | 3 | no |
| *yejM* | ACCATACCTG | 53.4 | 3 | no |
| *yhbV* | TTCATAGCTC | 46.8 | 2 | *murB^d,e^* |
| *yhhQ* | GTCATATTGT | 46.0 | 1 | no |
| *yigP* | GGCATTCCAC | 58.7 | 3 | no |
|  |  |  |  |  |

*^a^* *S.* Typhimurium gene name in parenthesis

*^b^* PNA used in this study

*^c^* Orthologues in *E. coli* and *S*. Typhimurium have different annotations of the start codon location. If the *E. coli* start codon is applied to both, there are 2 mismatches.

*^d^* Off-target gene reported to be essential in *S*. Typhimurium with 9 or 10 bp matches with the PNA

*^e^* Off-target gene reported to be essential in *E. coli* with 9 or 10 bp matches with the PNA

**Table S4.** *E. coli*-specific PNAs

| Gene | PNA sequence | *T*_m_ | Orthologue in *S.* Typhimurium | Off-target essential gene *E. coli* |
| --- | --- | --- | --- | --- |
| *alsK* | TGCATGAGGC | 65.6 | no | no |
| *bamA* | GCCATCGTTA | 56.0 | no | no |
| *bamD* | GTCATGACGT | 58.6 | no | *yidC* |
| *can* | TTCATGGAGG | 60.5 | no | *hemL* |
| *chpS* | CGCATCTGCA | 60.4 | no | no |
| *cohE* | TTCATAACGA | 52.6 | no | *lolD* |
| *dicA* | TCCATAGTTA | 46.3 | no | no |
| *fbaA* | GACATGTCTC | 52.4 | no | *rpmB* |
| *ispU* | ATCACGCGTT | 59.3 | no | no |
| *mazE* | ATCATAACCC | 49.7 | no | no |
| *racR* | AGCATTGCTT | 54.2 | no | no |
| *secM* | CTCACGTTAT | 47.7 | no | *rimN* |
| *yagG* | GTCATCAGAA | 56.3 | no | no |
| *yceQ* | GACACTCTGC | 57.1 | no | no |
| *ydfB* | TCCATATCAC | 46.5 | no | no |
| *yefM* | TTGACATGCG | 59.9 | no | no |

**Table S5.** Number of cells used for inoculation of mixed culture.

|  | No. of cells (1 x 10^4^) | | | |
| --- | --- | --- | --- | --- |
| Mixed culture | *B. subtilis* | *E. coli* | *K. pneumoniae* | *S. enterica* |
| *B. subtilis - K. pneumoniae* | 6 | - | 0.6 | - |
| *B. subtilis - S.* Typimurium | 6 | - | - | 0.7 |
| *E. coli - S.* Typimurium | - | 6 | - | 1 |
| *E. coli - K. pneumoniae* | - | 4 | 2.7 | - |
| *K. pneumoniae - S.* Typimurium | - | - | 4 | 3 |
| *B. subtilis - K. pneumoniae - S.* Typimurium | 4.7 | - | 1.3 | 0.7 |

**Table S6.** Oligonucleotides used in this study

| Oligo name | Target | Oligo sequence* (5’ -> 3’) |
| --- | --- | --- |
|  |  |  |
| ECpdf-F | *def* | cgagacgatgtacgcagaag |
| ECpdf-R |  | ggctttcagacgatccagtt |
| SEpdf-F | *def* | actcattaacccggaattgct |
| SEpdf-R |  | caggcggtcgagtttttcta |
| KPpdf-F | *def* | ctggagaaagatggcgaaac |
| KPpdf-R |  | aatgcgctgttgtttgagtg |
| BSpdf-F | *def* | ggaaacacctgcggaaacc |
| BSpdf-R |  | ctgcacggctcttgctaaaa |

**References**

**1.** **Roy A, Kucukural A, Zhang, Y** (2010) I-TASSER: a unified platform for automated protein structure and function prediction. Nature Protocols, 5: 725-738

**2. Söding J, Biegert A, Lupas AN** (2005) The HHpred interactive server for protein homology detection and structure prediction. Nucleic Acids Res., **33:**W244–8

**3. DeLano, WL (**2002) The PyMOL Molecular Graphics System. DeLano Scientific, San Carlos, CA, USA. http://www.pymol.org
